# Association between hospital-onset SARS-CoV-2 and ending universal admission testing and masking at five US hospitals

**DOI:** 10.1017/ash.2024.97

**Published:** 2024-09-16

**Authors:** Theodore Pak, Sanjat Kanjilal, Cara McKenna, Chanu Rhee, Michael Klompas

**Affiliations:** Massachusetts General Hospital; Harvard Medical School / Harvard Pilgrim Healthcare Institute; Harvard Pilgrim Healthcare Institute; Brigham and Women’s Hospital / Harvard Medical School; Harvard Medical School

## Abstract

**Background:** Many US hospitals have stopped universal masking and testing all patients on admission for SARS-CoV-2. We assessed the association of ending universal masking and admission testing with the incidence of hospital-onset SARS-CoV-2 infections in five Massachusetts hospitals. **Method:** We conducted a retrospective study of all patients admitted between March 6, 2020 and December 14, 2023 and identified hospital-onset SARS-CoV-2 infections (newly positive SARS-CoV-2 PCR tests >4d after arrival) and community-onset infections (newly positive ≤4d after arrival). We excluded cases if local infection control teams discontinued precautions within 4d (suggesting a false positive or remote/resolved infection). We calculated weekly ratios between hospital-onset and community-onset SARS-CoV-2 cases to account for changes in community SARS-CoV-2 incidence over time. We then performed interrupted time series analysis, looking for changes in the ratio of hospital-onset to community-onset cases across three periods: pre-Omicron period with universal testing and masking in place (March 6, 2020–Dec 16, 2021); Omicron period with universal testing and masking in place (Dec 17, 2021–May 11, 2023); and Omicron period without universal testing and masking (May 12, 2023–Dec 14, 2023). We performed medical record reviews on 100 randomly selected hospital-onset cases after May 12, 2023 to examine if community-onset cases were being misclassified as hospital-onset cases. **Result:** During the study period, there were 626,908 patient admissions, including 24,980 with community-onset and 1,510 with hospital-onset SARS-CoV-2 infections. The mean weekly ratio of new hospital-onset to community-onset SARS-CoV-2 infections rose from 2.6% before Omicron, to 8.5% (95% CI, 7.0–9.9%) during Omicron, to 17% (95% CI, 15–19%) after universal admission testing and masking ended (Figure 1). There was a significant immediate level change after the pre-Omicron-to-Omicron transition (140% relative increase; 95% CI, 40–240%) and after universal admission testing and masking ended (110% relative increase; 95% CI, 73–150%). On medical record review of 100 randomly selected hospital-onset SARS-CoV-2 cases after universal admission testing had ended, 89% had new symptoms at the time of testing, 80% had PCR cycle thresholds ≤30, 27% had a known COVID-19 exposure, and 97% met at least one of these criteria. In-hospital mortality occurred in 8% of the 100 reviewed cases. **Conclusion:** Stopping universal masking and admission testing of all hospitalized patients at five Massachusetts hospitals was associated with a significant increase in hospital-onset COVID-19. Nosocomial COVID-19 remains a common complication of hospital care. Preventing nosocomial infections in this vulnerable population remains an important safety goal.

**Figure:**
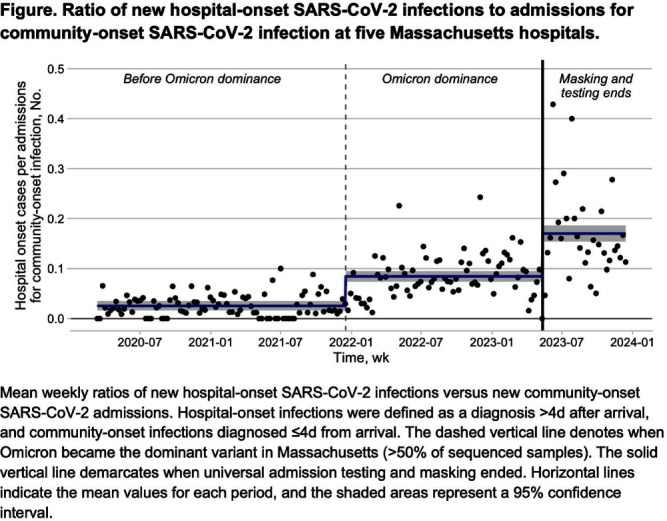

**Disclosure:** Theodore Pak: Founder/CEO - The East Harlem Software Company, Inc.

